# Decoding Interface Evolution: Toward Next-Generation Porous Carbon Supercapacitors via In-situ Characterization

**DOI:** 10.34133/research.1322

**Published:** 2026-07-08

**Authors:** Fang Yuan, Minjun Kim, Yiqiang Wu, Yusuke Yamauchi, Caichao Wan

**Affiliations:** ^1^ College of Materials and Energy, Central South University of Forestry and Technology, Changsha 410004, P. R. China.; ^2^ Australian Institute for Bioengineering and Nanotechnology (AIBN), The University of Queensland, Brisbane, QLD 4072, Australia.; ^3^ Department of Materials Process Engineering, Graduate School of Engineering, Nagoya University, Nagoya 464-8603, Japan.

## Abstract

The advancement of porous carbon supercapacitors is constrained by the inability of conventional characterization to probe their dynamic electrode–electrolyte interfaces. In-situ characterization bridges this gap, offering real-time observation of critical processes such as ion adsorption/desorption and surface reactions under operating conditions. This Perspective illustrates how these in-situ techniques are transforming mechanistic understanding, discusses persistent challenges including multimodal integration and spatiotemporal resolution trade-offs, and proposes a roadmap to translate these insights into the design of next-generation devices.

## Introduction

**Fig. 1.**
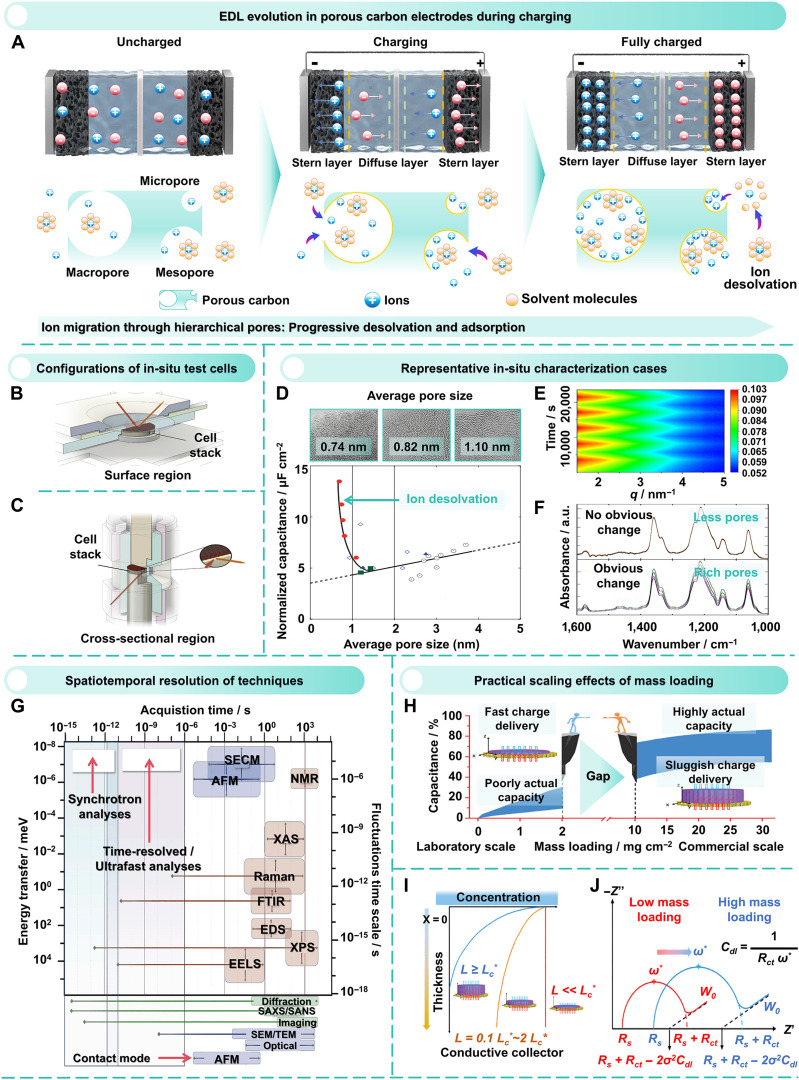
Decoding interface dynamics in porous carbon supercapacitors (SCs) through operando insights. (A) Evolution of the electric double layer (EDL) in porous carbon electrodes during charging. Upper panel: Ion migration during charging. Lower panel: Ion migration through hierarchical pores—progressive desolvation and adsorption. (B and C) Common operando cell configurations for (B) surface-sensitive and (C) cross-sectional analysis. Reproduced with permission from Ref. [8]. Copyright 2021, Elsevier. (D) Transmission electron microscopy (TEM) images and normalized capacitance of porous carbon materials (PCMs) with different pore sizes. Reproduced with permission from Ref. [10]. Copyright 2006, AAAS. (E and F) Representative in-situ characterization cases: (E) In-situ small-angle x-ray scattering (SAXS) revealing nanoscale pore evolution during ion insertion. Reproduced with permission from Ref. [14]. Copyright 2024, Electrochemical Society of Japan. (F) In-situ infrared (IR) spectroscopy confirming voltage-driven ionic response in pore-rich and pore-poor porous carbons. Reproduced with permission from Ref. [15]. Copyright 2013, American Chemical Society. (G) Spatiotemporal resolution of techniques, comparing acquisition time, energy domains, and characteristic time scales. Reproduced with permission from Ref. [8]. Copyright 2021, Elsevier. (H to J) Practical scaling effects: The influence of electrode mass loading on (H) capacitance, (I) electrolyte penetration depth, and (J) electrochemical impedance. Reproduced with permission from Ref. [18]. Copyright 2021, Royal Society of Chemistry. SECM, scanning electrochemical microscopy; AFM, atomic force microscopy; NMR, nuclear magnetic resonance; XAS, x-ray absorption spectroscopy; FTIR, Fourier transform infrared spectroscopy; EDS, energy-dispersive spectroscopy; XPS, x-ray photoelectron spectroscopy; EELS, electron energy-loss spectroscopy; SAXS, small-angle x-ray scattering; SANS, small-angle neutron scattering; SEM, scanning electron microscopy; TEM, transmission electron microscopy

Supercapacitors (SCs) store charge through fast ion adsorption at electrode–electrolyte interfaces (electric double-layer capacitance, Fig. 1A) and/or faradaic reactions (pseudocapacitance), offering exceptional power output, rapid kinetics, and long cycle life (typically 10^4^ to 10^6^ cycles) [1,2]. Porous carbon materials (PCMs) dominate SC electrodes due to their high surface area, tunable pore structures, and stability during cycling [3]. However, this long-term stability can be undermined by interfacial side reactions that escape conventional postmortem detection, motivating operando diagnostics. The growing interest in sustainable carbon sourcing, including the use of recycled graphite from spent batteries, further underscores the importance of understanding structure–performance relationships in carbon-based electrodes [4,5]. At the electrode level, the performance is governed by a complex interplay of microstructure and chemical features: Pore geometry dictates ion transport; carbon structural disorder affects charge storage and conductivity; and surface chemistry can introduce redox activity [6].

However, these processes occur dynamically at buried solid–liquid interfaces during operation, remaining largely inaccessible to conventional ex-situ characterization [7]. Real-time observation is therefore essential to link structure to function. While individual in-situ techniques have been reviewed in specialized contexts, a critical synthesis that identifies shared interpretive challenges across techniques and translates these collective mechanistic insights into actionable design principles has been lacking. This Perspective fills this gap. Rather than merely cataloging techniques, we examine what each method reveals about the evolving interface, diagnose cross-cutting bottlenecks that persist, and propose a roadmap toward predictive interface engineering for next-generation porous carbon SCs.

## Why In-situ Matters: Beyond the Limits of Ex-situ Characterization

Conventional ex-situ characterization fails to capture the dynamic, millisecond-scale processes (such as ion adsorption and pore evolution) and risks introducing artifacts during sample transfer, thereby obscuring true structure–property links.

In-situ methods overcome this by integrating electrochemical control with real-time probing within operational cells. This synchronizes performance metrics with structural data in a sealed, stable electrochemical environment. It minimizes transfer artifacts and directly links interfacial dynamics to device function. Employing either surface-sensitive or cross-sectional configurations (Fig. 1B and C), these techniques provide a faithful, real-time view of the working electrode–electrolyte interface, building critical correlations between nanoscale events and macroscopic behavior [8].

## Decoding the Interface: What In-situ Techniques Reveal

The capacitance of porous carbon electrodes is fundamentally governed by pore size and connectivity: Micropores provide abundant adsorption sites, mesopores facilitate ion transport, and their synergistic combination maximizes charge storage (Fig. 1D) [9,10]. Advanced in-situ techniques provide direct observation of mechanisms and degradation/recovery comparisons in porous carbons. The value of multitechnique electrochemical evaluation in establishing reliable performance metrics is widely recognized across the broader SC literature [11–13]. These techniques can be categorized into 3 complementary groups based on their core principles.

Spectroscopic techniques utilize electromagnetic waves to probe the evolution of nanostructure, chemical composition, and interfacial bonding. For instance, in-situ x-ray diffraction tracks crystallographic changes such as reversible interlayer expansion upon ion intercalation and the formation of electrolyte-derived crystalline phases, while in-situ x-ray photoelectron spectroscopy reveals voltage-driven asymmetric ion migration through shifts in cation-to-anion peak intensity ratios. In-situ small-angle x-ray scattering is particularly powerful for resolving nanoscale structural dynamics within pores (Fig. 1E), including pore expansion during ion uptake and ion rearrangement processes [14]. Complementary vibrational spectroscopies probe molecular-level responses: In-situ Raman spectroscopy correlates the D/G band intensity ratio with ion-adsorption-induced disorder and hydration shell restructuring, and in-situ infrared spectroscopy identifies voltage-dependent ion coinsertion mechanisms and surface oxidation pathways (Fig. 1F) [15]. In-situ small-angle neutron scattering further exploits contrast variation to illuminate ion solvation and distribution within subnanometer pores.

Electrochemical and magnetic resonance techniques monitor charge, spin, and local ionic environments during operation. In-situ electrochemical impedance spectroscopy, especially when coupled with distribution of relaxation time analysis, deconvolutes the contributions of charge transfer, ion adsorption, and bulk diffusion, providing a means to correlate PCM hydrophilicity with electrochemical performance. Complementarily, in-situ electron paramagnetic resonance spectroscopy quantifies the formation and stability of radical species as a function of potential, while in-situ nuclear magnetic resonance spectroscopy offers unique insights into the local environment and mobility of ions within pores, quantifying adsorbed species and probing ionic desolvation dynamics [16].

Gravimetric and gas analysis techniques establish a direct link between electrochemical reactions and mass transport. In-situ electrochemical quartz crystal microbalance monitors real-time mass changes to elucidate ion exchange stoichiometry and the role of solvation, revealing effects of interlayer spacing, electrolyte specificity, and ion solvation number. In-situ differential electrochemical mass spectrometry identifies and quantifies gaseous products (such as H_2_ and O_2_) on short time scales, clarifying parasitic reactions and electrolyte decomposition [17]. Its pulsed variant extends this capability to capture transient gas evolution on millisecond time scales, confirming surface oxidation processes in PCMs.

## From Bottlenecks to Roadmap: Navigating the In-situ Challenge

While pivotal for understanding charge storage, in-situ characterization of porous carbon SCs faces intrinsic bottlenecks. Technically, integrating complementary methods is hampered by incompatible operational environments (such as liquid vs. vacuum). Fundamentally, a spatiotemporal mismatch exists between fast, multiscale interfacial dynamics and the finite resolution of any single technique, creating observational blind spots (Fig. 1G). Analytically, interpreting multimodal data is complicated by signal overlap, risking mechanistic error. Practically, the transition to industry is hindered by a lack of standardized protocols and the performance gap between model lab electrodes and practical thick electrodes (Fig. 1H to J) [18]. These bottlenecks collectively relegate in-situ characterization to a diagnostic role—revealing what happens, but not how to design for it. Moving from observation to actionable prediction therefore requires a focused roadmap:

1. From incompatible environments to integrated platforms. The difficulty of combining techniques in disparate conditions (e.g., liquid vs. vacuum) calls for modular operando cells with smart environmental control that can host complementary probes within a single experiment. Synchronized multiprobe experiments, coupled with data fusion algorithms, further address the spatiotemporal resolution mismatch by stitching together partial views into a unified picture of interface dynamics.
2. From signal overlap to causal understanding. The analytical risk of mechanistic error arising from overlapping signals in multimodal datasets can be mitigated by building open databases that link dynamic structural and chemical descriptors to device performance. Machine learning trained on such data can then disentangle correlated signals, identify true performance determinants, and guide the predictive synthesis of tailored porous carbons.
3. From lab-scale observation to industrial translation. The value of multitechnique validation in establishing reliable performance benchmarks is well recognized in the broader SC literature [19,20]. Building on this, benchmark testing protocols—including standardized stability assessment under realistic cycling conditions—are needed to address the lack of cross-lab comparability and the performance gap between model electrodes and practical thick electrodes. Validated models that bridge these 2 scales by incorporating mechanistic insights from in-situ characterization into predictive, scalable simulations can position in-situ characterization as a cornerstone for rational device design and quality control.
